# Polymicrogyria: epidemiology, imaging, and clinical aspects in a population-based cohort

**DOI:** 10.1093/braincomms/fcad213

**Published:** 2023-08-11

**Authors:** Sintia Kolbjer, Daniel A Martín Muñoz, Anne K Örtqvist, Maria Pettersson, Anna Hammarsjö, Britt-Marie Anderlid, Maria Dahlin

**Affiliations:** Department of Molecular Medicine and Surgery, Karolinska Institutet, Stockholm 17177, Sweden; Department of Paediatric Neurology, Astrid Lindgren Children’s Hospital, Karolinska University Hospital, Stockholm 17176, Sweden; Department of Neuroradiology and Paediatric Radiology, Karolinska University Hospital, Stockholm 17176, Sweden; Clinical Epidemiology Division, Department of Medicine, Solna, Karolinska Institutet, Stockholm 17177, Sweden; Department of Obstetrics and Gynaecology, Visby County Hospital, Visby 62155, Sweden; Department of Molecular Medicine and Surgery, Karolinska Institutet, Stockholm 17177, Sweden; Department of Clinical Genetics, Karolinska University Hospital, Stockholm 17176, Sweden; Department of Molecular Medicine and Surgery, Karolinska Institutet, Stockholm 17177, Sweden; Department of Clinical Genetics, Karolinska University Hospital, Stockholm 17176, Sweden; Department of Molecular Medicine and Surgery, Karolinska Institutet, Stockholm 17177, Sweden; Department of Clinical Genetics, Karolinska University Hospital, Stockholm 17176, Sweden; Department of Paediatric Neurology, Astrid Lindgren Children’s Hospital, Karolinska University Hospital, Stockholm 17176, Sweden; Department of Women’s and Children’s Health, Karolinska Institutet, Stockholm 17177, Sweden

**Keywords:** prevalence, incidence, malformation of cortical development, microcephaly, epilepsy

## Abstract

Polymicrogyria is estimated to be one of the most common brain malformations, accounting for ∼16% of malformations of cortical development. However, the prevalence and incidence of polymicrogyria is unknown. Our aim was to estimate the prevalence, incidence rate, neuroimaging diversity, aetiology, and clinical phenotype of polymicrogyria in a population-based paediatric cohort. We performed a systematic search of MRI scans at neuroradiology department databases in Stockholm using the keyword polymicrogyria. The study population included all children living in the Stockholm region born from January 2004 to June 2021 with polymicrogyria. Information on the number of children living in the region during 2004–21 was collected from records from Statistics Sweden, whereas the number of births for each year during the study period was collected from the Swedish Medical Birth Register. All MRI scans were re-evaluated, and malformations were classified by a senior paediatric neuroradiologist. The prevalence and yearly incidence were estimated. Clinical data were collected from medical records. A total of 109 patients with polymicrogyria were included in the study. The overall polymicrogyria prevalence in Stockholm was 2.3 per 10 000 children, and the overall estimated yearly incidence between 2004 and 2020 was 1.9 per 10 000 person-years. The most common polymicrogyria distribution was in the frontal lobe (71%), followed by the parietal lobe (37%). Polymicrogyria in the peri-sylvian region was observed in 53%. Genetic testing was performed in 90 patients revealing pathogenic variants in 32%. Additionally, 12% had variants of uncertain significance. Five patients had a confirmed congenital infection, and in six individuals, the cause of polymicrogyria was assumed to be vascular. Epilepsy was diagnosed in 54%. Seizure onset during the first year of life was observed in 44%. The most common seizure types were focal seizures with impaired awareness, followed by epileptic spasms. Thirty-three of 59 patients with epilepsy (56%) were treated with more than two anti-seizure medications, indicating that pharmacoresistant epilepsy is common in polymicrogyria patients. Neurodevelopmental symptoms were observed in 94% of the individuals. This is the first population-based study on polymicrogyria prevalence and incidence. Confirmed genetic aetiology was present in one-third of individuals with polymicrogyria. Epilepsy was common in this patient group, and the majority had pharmacoresistant epilepsy. These findings increase our knowledge about polymicrogyria and will help in counselling patients and their families.

## Teaser

Kolbjer *et al.* report a polymicrogyria prevalence in a population-based paediatric cohort of 2.3 per 10 000 children and an overall yearly incidence rate at 1.88. The genetic cause was revealed in one-third of individuals. The most common polymicrogyria distribution was in the frontal lobe, and epilepsy occurred in 54%.

## Graphical Abstract

**Graphical Abstract:**
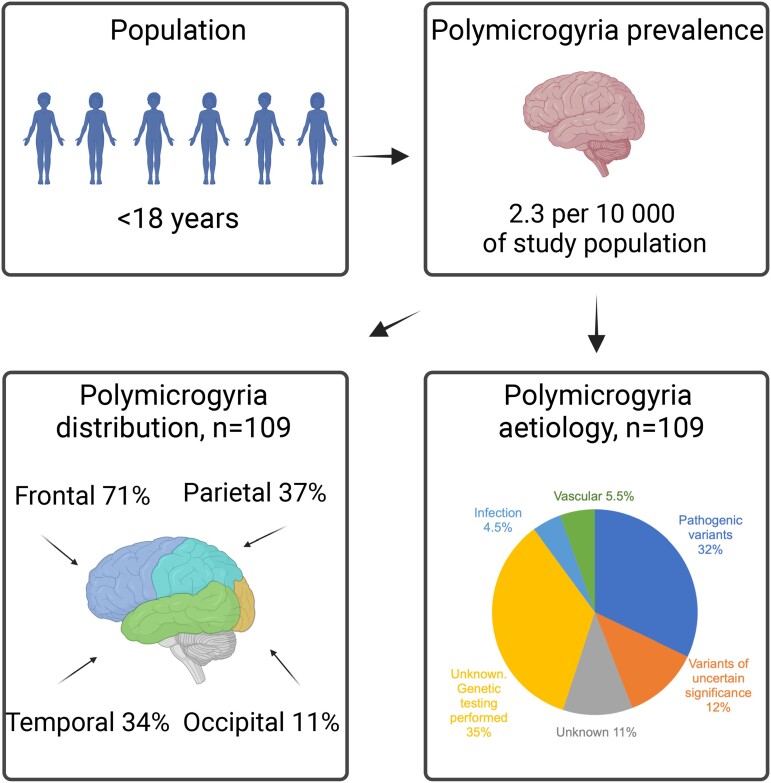

## Introduction

Among malformations of cortical development (MCDs), polymicrogyria (PMG) is estimated to be one of the most common brain malformations. The Leventer *et al.*^1^ study from 1999 on MCD showed data from a single centre with a catchment area of 4.4 million inhabitants. The study identified 109 patients with different MCDs of which 16% had PMG. However, the prevalence and incidence of PMG in population-based cohorts is insufficiently studied. Most PMG studies are case studies including small numbers of patients, but during recent years, multi-centre studies with larger PMG cohorts have been published.^2^

According to the international consensus recommendations provided by the MCD network Neuro-MIG, PMG is defined as an MCD with an excessive number of abnormally small cerebral gyri with cortical over-folding, irregular pebbled cortical surface and a stippled grey-white matter boundary.^3^ PMG can be isolated or associated with other brain malformations. According to the classification of MCD by Barkovich *et al.*,^4^ which is the most accepted and with the most recent update from 2012, PMG is a malformation due to abnormal post-migrational development. However, with new genetic information and PMG histopathology studies, discovering abnormalities in leptomeninges overlying the PMG cortex, the Neuro-MIG consortium has suggested a re-classification of PMG to the group of late migrational rather than post-migrational MCD.^5‐7^ PMG is divided into three groups: (i) PMG with transmantle clefts (schizencephaly) or calcifications, (ii) PMG without clefts or calcifications classified by location and (iii) syndromes with PMG. Along with the development of high-resolution imaging, molecular technologies and discovery of new genes and pathways, our knowledge of the underlying pathogenetic mechanisms of MCD has increased.

PMG is a highly heterogeneous malformation and can be caused by genetic, infectious, metabolic or vascular factors. The genetic causes of PMG include several microdeletion/microduplication syndromes, such as 1p36 deletion and 22q11.2 deletion syndromes, as well as single gene or pathway disorders.^8^ During recent years, advances in genetic testing have revealed more than 50 genes associated with PMG.^9^ Nevertheless, the diagnostic yield of genetic testing in PMG is low, in different studies between 12 and 20%.^10‐12^ Several congenital infections cause PMG, and congenital cytomegalovirus (CMV) infection appears to be the most significant.^13^ Other congenital infections associated with PMG are toxoplasmosis, varicella and Zika virus.^14^ Among metabolic causes, the most frequent disorders are peroxisomal.^15^ Additionally, there are several syndromes with PMG, of which many have a genetic aetiology.^10^

In Stockholm County, there are two hospitals with departments for paediatric neurology. Here, in-patient and out-patient service is given to children with neurodevelopmental disorders and/or epilepsy. All MRI brain scans with pathological findings performed in Stockholm County, even if they are performed at other radiological departments, are sent to these hospitals for re-examination by paediatric neuroradiologists. By patient selection from these two neuroradiology clinics, we included all children diagnosed with PMG in the Stockholm area. Thus, we were able to present a clinically and radiologically well-characterized, population-based cohort. Our aim was to study the prevalence and incidence of PMG and perform a detailed radiologic and clinical characterization of the patients.

## Materials and methods

### Study population

We performed a systematic search of MRI scans at the two paediatric neuroradiology department databases in Stockholm at Astrid Lindgren Children’s Hospital at Karolinska University Hospital, Solna and Huddinge and at Sachsska Children’s Hospital at Södersjukhuset. All children born from January 2004 to June 2021 with MRI performed before October 2021 were included in our cohort. We defined the age for PMG diagnoses as the age when the MRI examination, which led to the diagnosis, was performed, although clinical symptoms could have been present days or even months before neuroimaging. The keywords used were PMG, schizencephaly and pachygyria. We included patients with schizencephaly only if there were areas with PMG. Pachygyria was included only in the first search for clinically diagnosed patients.

A total of 174 MRI examinations of 127 individuals were included at the first evaluation. Imaging was acquired on 3 and 1.5 T Siemens (Prisma, Skyra, Trio Tim, Verio, Aera, Trio Tim, Symphony Tim and Avanto), Philips Healthcare (Ingenia, Intera, Achieva and Panorama) and GE Medical Systems (Discovery MR750w, Signa HDxt and Optima MR450w). Fifty-one patients had imaging from 1.5 T, while the remaining 58 had either 3 T or both 1.5 and 3 T scanning. At re-evaluation, 18 individuals were not included in the study as MRI showed pachygyria or schizencephaly without PMG. Also, patients with dysgyria were included only if co-existing with PMG. All clinical data were collected between September and November 2021. The mean age of individuals at data collection was 9.3 years. The youngest included person was 0.5 years old, while the oldest was 17.4 years old. Demographic data on the population were collected from Statistics Sweden, whereas the number of births for each year during the study period was collected from the Swedish Medical Birth Register.

### Neuroimaging classification

All MRI scans were re-evaluated, and malformations were classified by the same senior paediatric neuroradiologist. A few patients had several MRI investigations at different ages. PMG was classified based on topographic patterns used by Leventer *et al.*^2^, dividing PMG into six categories according to the major distribution: peri-sylvian, generalized, PMG with periventricular nodular heterotopia (PNH-PMG), frontal, parasagittal parieto-occipital and other. Peri-sylvian PMG was defined as PMG with a strict involvement of gyri around the sylvian fissure. In the PNH-PMG group, we allocated all individuals who had PNH, even if only small amounts of heterotopia were present. This group was then divided into subgroups according to the most salient PMG. The group ‘other’ included multi-focal, superior parasagittal, peri-sylvian PMG with contralateral schizencephaly, PMG with cleft and focal PMG. Associated malformations in corpus callosum and posterior fossa, as well as white matter changes, were registered. Also, an additional classification was performed dividing PMG according to affected lobes and regions, not only topographic patterns with major distribution. PMG was assumed to have a vascular cause if unilateral, deeply infolded, heterotopic grey matter brain malformation with areas of PMG surrounded by otherwise normal appearing brain was present. This could be suggestive of a focal vascular insult during early the pre-natal period with a disruption of migration and organization. The lack of anomalous vessels and residual haemorrhage or calcifications further supports the early timing in development with immune response and adaptation.

### Clinical data

From medical records, we collected clinical data, including perinatal data, ethnicity, occipitofrontal head circumference (OFC) at birth and at data collection, symptoms leading to neuroimaging, age at PMG diagnosis counting as date for MRI examination, presence of seizures and age at seizure onset, presenting seizure type, seizure and epilepsy type. Seizures were classified according to The International League Against Epilepsy (ILAE) seizure classification system in 2017.^16^ Epilepsy was defined as having experienced at least two unprovoked seizures or one unprovoked seizure and at least 60% risk for recurrent seizures, according to current ILAE classification of the epilepsies.^17^ Additionally, data on the number and EEG results of performed EEGs, number of used anti-seizure medications and other used anti-seizure treatments, cognitive and motor development as well as data on aetiological investigations were collected. Data on CMV testing using CMV PCR analysis in new-born urine or paired serology in the mother and child were available in 19 individuals. Microcephaly was defined as the OFC ≤−2 SD below the mean for age and sex. Severe microcephaly was defined as ≤3 SD below the mean. Macrocephaly was defined as the OFC ≥2 SD. Data on cognitive functions and presence of neurodevelopmental disorders, such as autism spectrum disorder and attention deficit hyperactivity disorder, were collected from the results of evaluations made by neuropsychologists and paediatric neurologists. All children with motor disorders were assessed by a physiotherapist. Data were collected on motor function examination performed by physiotherapists and paediatric neurologists.

### Genetic analysis

The data on genetic evaluation were collected from patients’ medical records. Patients were investigated using chromosome analysis, oligonucleotide array comparative genomic hybridization, multiplex ligation-dependent probe amplification, Sanger sequencing and exome sequencing (ES) or genome sequencing (GS). In cases without confirmed genetic or infectious aetiology, genetic testing or re-evaluation of previously performed analysis was offered. A total of 67 individuals were sequenced using GS (43 singleton and 24 trios) and 10 with ES (4 singletons and 6 trios). Genomic DNA derived from whole blood was sequenced using the Illumina Hiseq X Ten and NovaSeq 6000 platforms, using 30× a polymerase chain reaction (PCR)-free paired-end GS protocol and Agilent SureSelect Human All Exon V5 ES protocol. Detailed workflow for clinical GS at our centre has been described previously, and the same variant calling pipeline for single nucleotide variants was used for ES as for GS.^18,19^

### Statistical analysis

The prevalence, showing the proportion of cases in the population at a given time, was estimated by dividing the total number of children identified with PMG in Stockholm between 2004 and 2021 (*n* = 109) with the mean total number of children (0–17 years of age) in Stockholm for the same time period (*n* = 466 500). The number of children living in Stockholm for each included year was collected from publicly available demographic data from Statistics Sweden.^20^

The overall incidence rate, measuring the frequency with which a disease occurs over a specified time period, was estimated by dividing the number of cases of PMG born in Stockholm (*n* = 89) between 2004 and 2020 for each birth year with the number of births in Stockholm for each corresponding year. We also calculated the period incidence rate for three different time periods (2004–09, 2010–15 and 2016–20). The number of births for each year was collected from publicly available statistics from the Swedish Medical Birth Register.^21^ Individuals born in 2021 (<5) were not included as there was no publicly available data on number of births in Stockholm for 2021.

Descriptive statistics of clinical data are presented. To determine potential statistical differences of age at first MRI, age at first seizure, and epilepsy in relation to gender Mann–Whiney U-test was performed. Results were considered statistically significant if *P* < 0.05. Analyses were conducted in Stata version 15 (StataCorp, College Station, TX, USA).

The study was approved by the Regional Ethics Committee Stockholm (Dnr: 2021-01888).

## Results

A total of 109 patients with PMG were included in the study, whereof 67 (61%) were males (Table 1). Approximately one-third of the study cohort had non-Caucasian ethnicity, reflecting a multi-ethnical study population.

**Table 1:** Cohort characteristics of all 109 cases of PMG identified in Stockholm between 2004 and 2021

Variable	*n* = 109	100%
Sex		
Male	67	61.5
Female	42	38.5
Born in Sweden	97	89
Born in Stockholm	91	83.5
Pre-term	12	11
Twins	8	7.3
Age at PMG diagnosis (MRI) in years		
Median (IQR); min–max	1.3 (0.3–3); 0–15.4	
Mean (SD)	2.6 (3.4)	
Age at data collection in years		
Median (IQR); min–max	9.1 (5.9–12.5); 0.5–17.4	
Mean (SD)	9.3 (4.6)	
OFC		
At birth		
Microcephaly (≤−2 SD)	15	13.8
Severe microcephaly (≤−3 SD)	5	4.6
Normocephaly (>−2 to <2 SD)	62	56.9
Macrocephaly (≥2 SD)	15	13.8
Missing information	17	15.6
At data collection		
Microcephaly (≤−2 SD)	41	37.6
Severe microcephaly (≤−3 SD)	25	22.9
Normocephaly (>−2 to <2 SD)	50	45.9
Macrocephaly (≥2 SD)	10	9.2
Missing information	8	7.3
Seizures		
Yes^a^	66	60.6
No	43	39.4
EEGs performed		
Yes	79	72.5
Neurodevelopmental disorders		
Cognition		
Developmental delay	17	15.6
Intellectual disability	58	53.2
Mild intellectual disability	12	11
Moderate intellectual disability	8	7.3
Severe intellectual disability	22	20.2
Intellectual disability, unspecified	16	14.7
Learning difficulties	<5	N/A
No cognitive disabilities	31	28.4
ASD	16	14.7
ADD/ADHD	16	14.7
Motor function disorders	67	61.5
Cerebral palsy	36	33
Unilateral spastic	18	16.5
Bilateral spastic Dyskinetic, mixed type	15 <5	13.8 N/A
Neurodevelopmental motor disorders	23	21.1
Hypotonia	8	7.3
At least one neurodevelopmental disorder	103	94.5
Cooccurence of at least 2 neurodevelopmental disorders	71	65.1
No neurodevelopmental disorders	6	5.5

a Includes seven (6.4%) patients who only had febrile seizures.

ASD, autism spectrum disorder; ADD, attention deficit disorder; ADHD, attention deficit hyperactivity disorder; N/A, not applicable; SD, standard deviation.

### Prevalence and incidence

**Figure 1:**
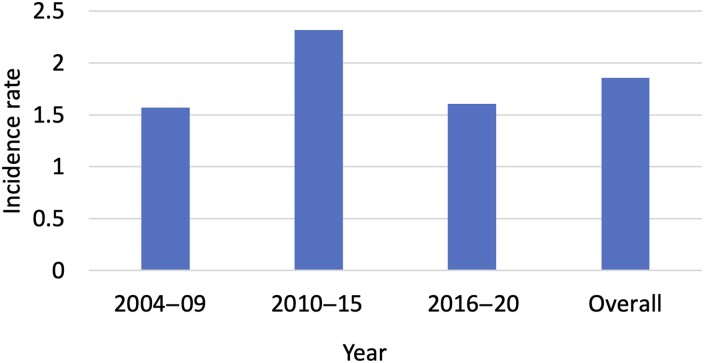
Period incidence rate per 10 000 person-years.

The overall prevalence of PMG among children in Stockholm was 0.02% [2.31 (95% CI 1.90–2.80) per 10 000]. The overall estimated yearly incidence of PMG between 2004 and 2020 was 1.88 (95% CI 1.51–2.31) per 10 000 person-years. The estimated incidence rates of PMG for the years 2004–09, 2010–15 and 2016–20 were 1.63 (95% CI 1.06–2.41), 2.32 (1.66–3.15) and 1.60 (1.02–2.41) per 10 000 person-years, respectively (Fig. 1).

Seven of the patients in the study cohort passed away at a mean age of 19 months (median 5 months; range 1–94 months). Of these, three had known genetic diagnoses with mutations in *PEX1* (syndrome), *TUBA1A* and *PEX2*, respectively. In the remaining four patients, a genetic diagnosis could not be revealed despite ES trio analysis. One of them had hemimegalencephaly with PMG. Three of the patients had consanguineous parents.

### Neuroimaging

The results of PMG patterns are given in Table 2. Of the 109 patients, PNH-PMG was the most common pattern, counting for 24% (*n* = 26) followed by peri-sylvian PMG in 21% (*n* = 23), generalized PMG in 20% (*n* = 22) and frontal PMG in 19% (*n* = 21). Many patients had a mix of different patterns due to overlapping distribution and gradient of severity. For example, peri-sylvian PMG could have a more extensive distribution in one hemisphere and only multi-focal on the contralateral side, while a clear parasagittal parieto-occipital PMG also could have a small focus of PMG in the sylvian fissures.

**Table 2:** PMG patterns and PMG distribution as well as associated abnormalities in the study cohort

PMG pattern	Total, *n* (%)	Bilateral, *n*			Unilateral, *n*		
		All	Symmetric	Asymmetric	All	Right	Left
Peri-sylvian	23 (21)	20	11	9	3	1	2
Generalized	22 (20)	20	20	0	2	1	1
PNH-PMG	26 (24)	24	1	1	2	1	1
Peri-sylvian	5 (5)	5	5	0	0	0	0
Global	7 (6)	7	4	3	0	0	0
Frontal	9 (8)	8	6	2	1	0	1
Frontoparietal	2 (2)	2	0	2	0	0	0
Posterior	3 (3)	2	2	0	1	1	0
Frontal	21 (19)	16	11	5	5	2	3
BPPP	2 (2)	2	0	2	0	0	0
Other	15 (14)	11	4	7	4	1	3

PMG distribution	Total, *n* (%)	Bilateral, *n*			Unilateral, *n*		
		All	Symmetric	Asymmetric	All	Right	Left
Frontal	77 (71)	61	37	24	16	6	10
Parietal	40 (37)	31	22	9	9	4	5
Temporal	37 (34)	31	21	10	6	3	3
Occipital	12 (11)	11	4	7	1	0	1
Global	17 (16)	14	14	0	3	1	2
Peri-sylvian	58 (53)	53	37	16	5	3	2
Inter-hemispheric	20 (18)	15	9	6	5	2	3

Associated abnormalities	Total, *n* = 109 (%)
White matter	
Abnormal high signal	26 (24)
Reduced volume	41 (38)
Corpus callosum	
Hypoplasia	24 (22)
Dysplasia	8 (7)
Agenesis	9 (8)
Hypertrophy	4 (4)
Normal	65 (60)
Cerebellar hypoplasia	
Generalized	5 (5)
Hemisphere	1 (1)
Vermis	3 (3)
Calcifications	9 (8)

BPPP, bilateral parasagital parieto-occipital PMG.

**Figure 2:**
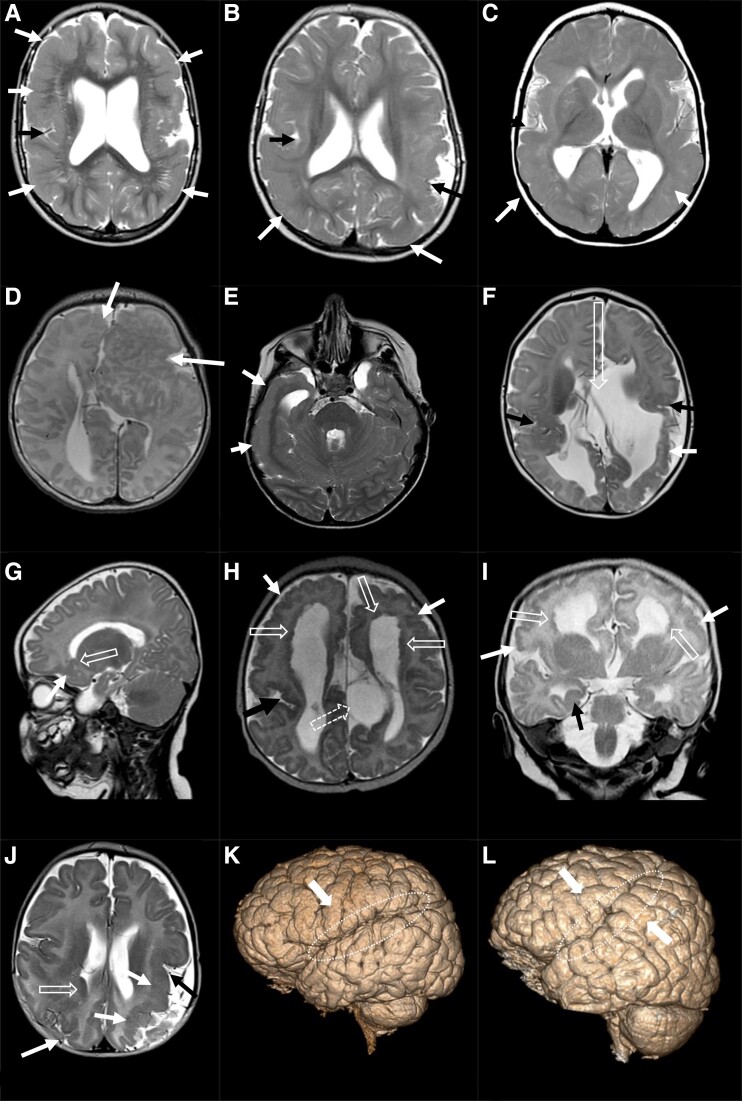
**MRI features.** (**A**–**C**) Axial T2 of PMG (solid arrows) with peri-sylvian distribution in three individuals with CMV infection. Black arrows for sylvian fissure. White arrows for PMG. (**A**) Bilateral peri-sylvian distribution, more severe left then right, with bilateral frontal and less parietal extension with effacement of sulci. Left sylvian fissure opened due to more extensive PMG; (**B)** bilateral peri-sylvian with predominantly posterior extension with more marked extension in the left than the right parietal lobe causing widening of left subarachnoid space; (**C**) with parietal and occipital distribution, arrows on superior occipital gyri. (**D**–**F**) Axial T2 of PMG after probable pre-natal vascular insult. (**D**) Left frontal dysgyria and PMG with a deeply infolded heterotopic grey matter brain malformation in the anterior left frontal lobe with anomalous subarachnoid spaces and vessels (not present on the image) with focal PMG also in the right frontal lobe (GS performed, unknown aetiology); (**E**) right temporal PMG with white matter volume loss and dilated temporal horn of the lateral ventricle (*NPHP1*); (**F**) bilateral peri-sylvian PMG (black arrows) with parasagittal volume defect (open arrow) and posterior white matter volume loss with PMG in the left inferior parietal lobe (genetic evaluation not performed). (**G**–**J**) T2 of sub-ependymal heterotopias (open arrow) and overlying PMG (white arrows). (**G**) Sagittal T2 with sub-ependymal heterotopia in left frontal horn with PMG in orbital gyri (*PTPN23*); (**H**) axial T2 with sub-ependymal heterotopia in bilateral frontal horns of lateral ventricles with PMG in frontal lobes. Right-sided peri-sylvian PMG (black arrow) and left parietal parasagittal cyst displacing the left thalamus inferiorly (dotted opened arrow); (**I**) Cor T2 of the same patient as in **H** with right-sided incomplete hippocampal inversion (globular shape, black arrow). Bilateral PMG (white arrows) with sub-ependymal heterotopia (open arrows) (Aicardi syndrome, GS trio with normal results); (**J**) sub-ependymal heterotopia in right trigonal part of lateral ventricles (open arrow on right side) with PMG in right parietal (white arrow). Left peri-sylvian PMG (black arrow) with left frontal and parietal distribution (white arrows) (parents declined genetic evaluation, CMV excluded). (**K** and **L**) 3D volume of right hemisphere with sylvian fissure (dotted circle). (**K**) Posterior ascending ramus of sylvian fissure extending horizontally into inferior parietal lobe with frontal PMG (white arrow) (*PIK3CA*); (**L**) horizontal ramus projects steep into ascending ramus of the sylvian fissure with a near vertical extension into the superior parietal lobe with peri-sylvian and peri-opercular PMG (white arrows) (GS performed, unknown aetiology).

PMG classification according to distribution is given in Table 2. The most common PMG distribution was frontal, seen in 77 (71%) cases. While most findings were bilateral and symmetrical in distribution, an anterior severity gradient of PMG was more common than an even distribution. Right- and left-sided gradients were slightly more common in the left hemisphere (23%; *n* = 25) than the right (20%; *n* = 22) in the whole group. The sylvian fissure was open anteriorly in 22% (*n* = 24), and a longer extension of the posterior ramus of the sylvian fissure into the parietal lobe was seen in 29% (*n* = 32) (Fig. 2). As part of the developmental changes, true hippocampal inversion was seen in 10 (9%), while more subtle changes with only globular-shaped hippocampus was seen in 21 (19%) cases. Only six (6%) cases had unilateral involvement, on the left side in four (4%) and two (2%) in the right side. PMG-associated central nervous system abnormalities are given in Table 2. Abnormal findings in the corpus callosum were present in 44 (40%) cases with hypoplasia in 24 (22%), followed by agenesis in 9 (8%) and dysplasia in 8 (7%) cases, posterior more common than diffusely. The cerebellum was affected in 9 (8%) cases, more commonly diffusely, and the brainstem only in 10 (9%). Pons was more often affected than the brainstem in general.

### Age and presenting symptom at PMG diagnosis

**Figure 3:**
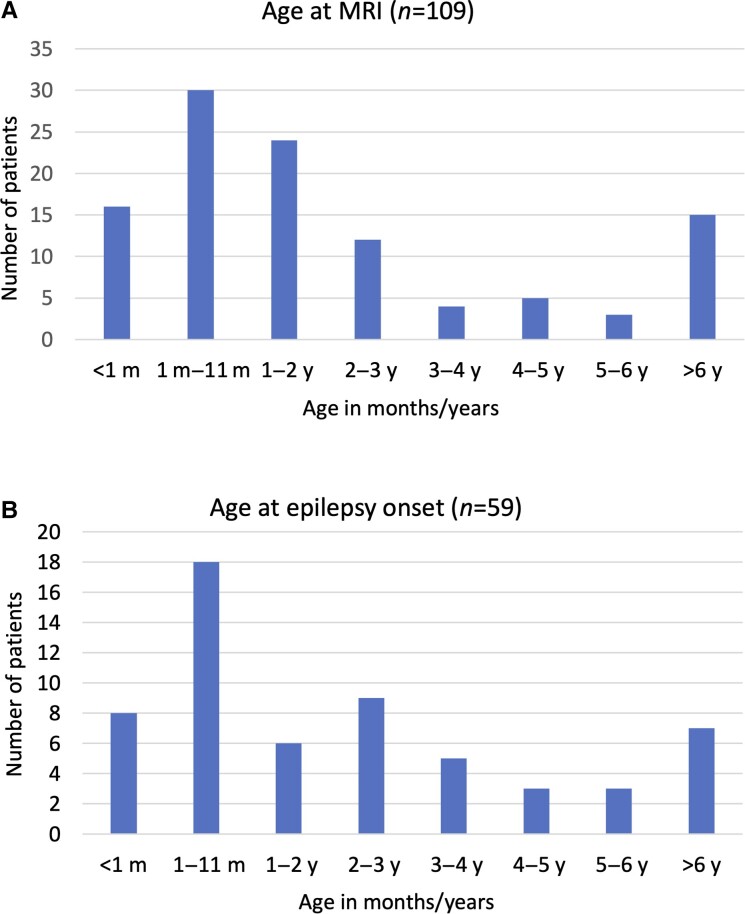
**PMG diagnosis and epilepsy onset in relation to age.** (**A**) Age at PMG diagnosis. (**B**) Age at epilepsy onset. m, months; y, years.

The first MRI, which led to PMG diagnosis, was performed at the median age of 1.3 years [interquartile range (IQR) 0.3–3]. There was no statistical difference between males and females regarding age at MRI (*P* = 0.08). Forty-two per cent of the patients were diagnosed with PMG during the first year of life and 64% during the first 2 years of life (Fig. 3A). Notably, patients with bilateral PMG had more severe symptoms leading to diagnosis of PMG in 47% of cases during the first year of life compared with only in 33% in unilateral PMG. In general, PMG diagnoses were set earlier if the presenting symptoms were seizures, pre-natal symptoms or congenital malformations. The longest duration from first symptoms to neuroimaging was in the cases where the presenting symptom was developmental delay. The most frequent symptoms leading to MRI and diagnosis of PMG were seizures and developmental delay counting for 28 and 24%, respectively.

### PMG aetiology

Genetic testing was performed in 90 of 109 patients revealing a confirmed or presumed genetic aetiology in 35 (32% of the total cohort and 39% of the tested population). Of these, 6 (17%) had chromosomal aberrations and 29 (83%) pathogenic single-gene variants. Additionally, 13 (12%) of the genetically investigated patients had variants of uncertain significance (Supplementary Table 1). In 41 patients, we could not reveal a genetic cause of PMG despite genetic investigations including GS or ES with *in silico* gene panels in 21 cases (20 GS and 1 ES), ES trio analysis in one and GS trio analysis in 17. Two patients had undergone genetic testing with chromosomal microarray, excluding large copy number variants or aneuploidies, but had not been subjected to ES or GS. Congenital CMV was excluded in seven of these individuals.

Nineteen patients had not undergone genetic evaluation. Among these individuals, two were diagnosed by neuroimaging performed due to symptoms not related to PMG (minor head injury and transient sixth cranial nerve palsy), and otherwise healthy and therefore genetic evaluation was not performed. Five patients had a confirmed congenital infection, whereof three had congenital CMV, one congenital toxoplasmosis and one congenital herpes simplex virus 2. Additionally, two individuals had declined genetic testing, and in eight cases, blood samples could not be obtained.

In six individuals, the PMG cause was assumed to be vascular, based on the MRI findings. Genetic testing was performed in four of them. One individual had genetically confirmed Beckwith–Wiedemann syndrome (hypomethylation in IC2), but imaging strongly indicated pre-natal stroke, and PMG is not known to be associated with Beckwith–Wiedemann syndrome. The other three were analysed with GS with normal results. All six individuals with assumed vascular cause had unaffected pre-natal and perinatal history. Furthermore, one additional individual with presumed vascular aetiology had a pathogenic variant in *COL4A1* and therefore was classified as genetic.

**Figure 4:**
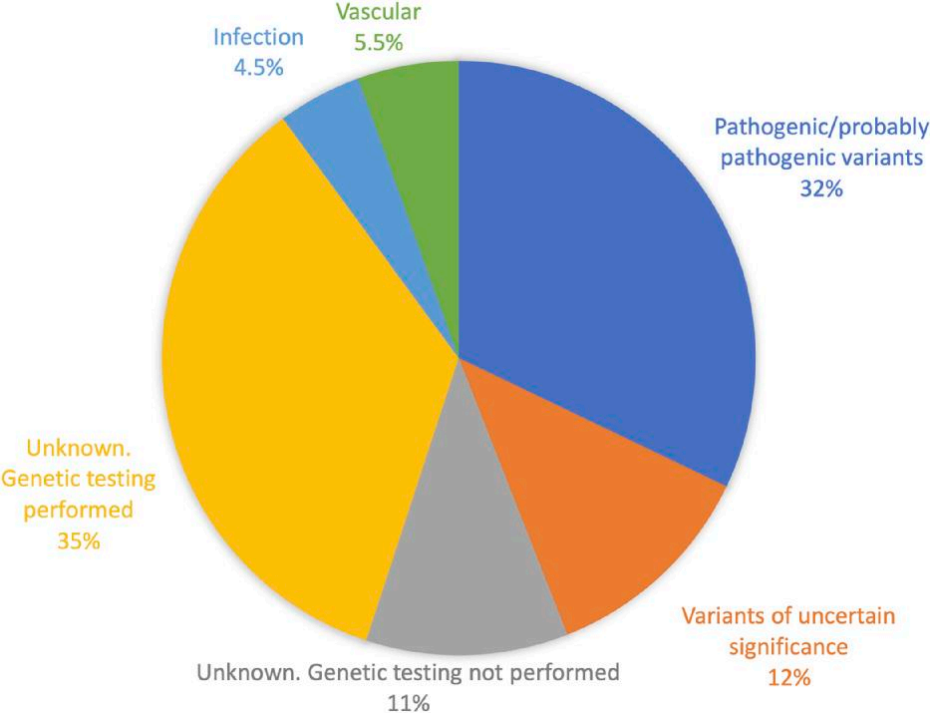
PMG aetiology in our cohort of 109 individuals.

Among the patients who were genetically tested without pathogenic variants, vascular or infectious aetiologies cannot be excluded. Overall, PMG aetiology was confirmed or presumed in 40 (36.7%) and strongly assumed in an additional 6 (5.5%) vascular cases. Thus, we have a confirmed or strongly suggested PMG aetiology in 46 (42%) cases (Fig. 4).

### Pre- and perinatal data

Most children were born at term (≥37 to <42 weeks of gestation). Data on gestational week at birth were documented in 107 patients but could not be obtained for 2 patients born in other countries. Only 12 (11%) patients were born pre-term, between gestation Weeks 30 and 36, and 4 (4%) post-term. Eight children were born from twin pregnancies, two of them preterm and one following the death of the co-twin. Among eight individuals born from twin pregnancies, two had pathogenic single-gene variants, one a variant of uncertain significance, two had genetic evaluation with normal results and three were not genetically tested. None of the presumed vascular cases were born from twin pregnancies.

### Occipitofrontal circumference

**Figure 5:**
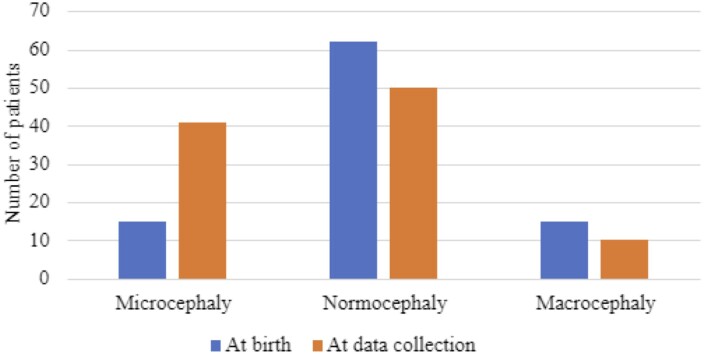
The OFC at birth (*n* = 92) and at time of data collection (*n* = 101).

Data on OFC at birth were available in 92 patients. Congenital microcephaly was observed in 15 (14%) patients of which 5 (33%) had severe microcephaly. At data collection, documented OFCs were available in 101 patients. In five patients, the OFC data were not available, neither at birth nor later. Forty-one (38%) individuals had microcephaly at follow-up, and of those, 25 (61%) had severe microcephaly. Thirteen of 15 patients with primary microcephaly remained microcephalic, while two patients with OFC −2 SD at birth became normocephalic. Twenty-one patients (50%) developed secondary microcephaly. In seven patients with microcephaly, we could not obtain data on birth OFC and could therefore not determine if they had primary or secondary microcephaly. Fifteen (14%) patients presented with macrocephaly at birth, while 10 (9%) had macrocephaly at data collection. Data on OFC are presented in Table 1 and Fig. 5.

### Neurodevelopmental symptoms

Neurodevelopmental disorders were observed in 103 (94%) individuals (see Table 1). Cooccurrence of two or more neurological comorbidities other than epilepsy was observed in 71 (65%) individuals. Seventy-eight (72%) had cognitive impairment, and of those, 58 (53%) had intellectual disability and 20 (19%) developmental delay or learning difficulties. Autism spectrum disorder and attention deficit disorder or attention deficit hyperactivity disorder was diagnosed in 16 (15%) individuals each. We could not observe a statistically significant difference in any neurodevelopmental disorder comparing the PMG group with epilepsy and the group without epilepsy (Supplementary Table 2).

A motor function disorder was present in 67 (61%) patients. Of these, 36 (33%) children had cerebral palsy, divided equally between unilateral and bilateral cerebral palsy (bilateral spastic, dyskinetic and mixed type; see Table 1). Other motor disorders, such as hypotonia, apraxia, developmental coordination disorder and poorly differentiated control, were present in 31 (28%) individuals. In our cohort, 19 individuals (17%) were non-ambulatory.

Only six individuals did not have any cognitive impairment or motor disorder. In four of these individuals, seizures were the presenting symptom leading to PMG diagnosis. In one case, PMG was diagnosed following a minor head injury leading to emergency head CT and later MRI, and in another patient, MRI was performed due to a transient sixth cranial nerve palsy.

### Seizures

Seizures occurred in 66 (61%) patients. In seven of them, only single or recurrent febrile seizures were observed at data collection. Epilepsy was diagnosed in 59 (55%) patients, of which 27 (46%) were females and 32 (54%) males (Table 3). In the total cohort, 27 of 41 (66%) females developed epilepsy in contrast to 32 of 67(48%) males (*P* = 0.07).

**Table 3:** Epilepsy characteristics of the study population

Epilepsy	*n* = 59	100%
Age at seizure onset (years)		
Median (IQR); min–max	1.6 (0.3–3.4); 0–11.4	
Mean (SD)	2.5 (2.9)	
Seizures		
Presenting seizure type		
Focal impaired awareness seizures	16	27.1
Epileptic spasms	7	11.9
Febrile seizures	7	11.9
Focal aware seizures	6	10.2
Other^a^	23	40
Number of seizure types		
median (IQR); min–max	2 (1–3); 1–6	
1	16	27.1
2	22	37.3
3	13	22
≥4	8	13.6
Seizure types		
Epileptic spasms	14	23.7
Focal aware seizures	9	15.3
Focal impaired awareness seizures	29	49.2
FBTCS	12	20.3
GTCS	6	10.2
Myoclonic seizures	7	11.9
Status epilepticus	11	18.6
Tonic seizures	11	18.6
EEG		
Performed EEG in our hospitals		
Yes	58	98.3
Number of EEGs		
Mean (SD)	6.7 (7.9)	
Median (IQR); min–m max	4 (2–8); 0–44	
Epileptiform activity		
Focal	21	35.6
Multi-focal	17	28.8
Generalized	<5	N/A
Combined^b^	14	23.7
Without epileptiform activity	6	10.2
Treatment		
Number of individuals with ASM	59	100
Number of previous and ongoing ASM		
Median (IQR); min–max	3 (2–6); 1–13	
Mean (SD)	4.2 (3.2)	
Pharmacoresistant, >2 ASM	33	55.9
Ketogenic diet	7	11.9
VNS	<5	N/A

a Atonic seizures, FBTCS, focal non-motor autonomic seizures, focal non-motor behaviour arrest, GTCS, myoclonic seizures, status epilepticus, and tonic seizures.

b Focal and multi-focal, focal and generalized, and multi-focal and generalized.

ASM, anti-seizure medications; FBTCS, focal to bilateral tonic clonic seizures; GTCS, generalized tonic clonic seizures; N/A, not applicable; SD, standard deviation.

Seizure onset occurred during the first year of life in 26 (24%) patients (Fig. 3B). The median age at first seizure was 1.6 years (IQR 0.3–3.4). For females, the median age of seizure onset was 0.4 years (IQR 0.2–4.1) compared with 2.1 years (IQR 0.6–3.3) for males. However, no statistically significant gender difference was found (*P* = 0.17).

The presenting seizure type varied, and the most common was focal seizure with impaired awareness, followed by epileptic spasms and focal aware seizures. Five patients (8%) had status epilepticus as the presenting seizure type. In six patients (10%), single or recurrent febrile seizures were observed prior the first unprovoked seizure. The epilepsy type was focal epilepsy in a majority of patients (*n* = 47 or 80%), seven patients (6%) had generalized epilepsy and five (5%) unclassified epilepsy with both focal and generalized seizures.

Epilepsy developed approximately in half of individuals with unilateral and bilateral PMG. However, individuals with bilateral PMG had earlier seizure onset, the majority (58%) during the first 2 years of life. In unilateral PMG, only 36% had seizure onset before the age of 24 months. Individuals with generalized PMG and PMG with peri-nodular heterotopia presented with generalized seizures or combination of focal and generalized seizures. We could not determine any significant correlation between lobar distribution of PMG and more frequent development of epilepsy. However, patients with occipital PMG had slightly less frequent development of epilepsy compared with PMG involving other lobes (Supplementary Table 3).

All patients with seizures performed EEG recordings; however, in one patient, EEGs were performed in another hospital. The number of EEG recordings varied between 1 and 40. EEG findings did not correlate constantly with PMG location as both patients with unilateral as well as bilateral PMG could have focal, multi-focal or combination of multi-focal–generalized epileptiform activity in different recordings. In addition, different EEG recordings in the same individual could show a variation in pattern.

Anti-seizure medications (ASMs) were used in 61 patients—in all 59 patients with epilepsy, in one patient with recurrent febrile seizures and in one patient without seizures but with multi-focal epileptiform activity and developmental delay. The patients had been treated with 1–13 different ASMs (mean 4, median, 3). Thirty-three of 59 patients with epilepsy (56%) were treated with more than two ASMs, indicating that pharmacoresistant epilepsy is common in PMG patients. In addition, eight patients had additional treatments; four had been treated with the ketogenic diet, one patient with vagus nerve stimulation (VNS) and three had both ketogenic diet and VNS. None of the patients in our cohort had undergone epilepsy surgery.

## Discussion

This is the first population-based study on incidence and prevalence in symptomatic PMG in children. Asymptomatic individuals will probably not come to medical attention or present only accidently, and the diagnosis of PMG could thus be a coincidental finding. This was the case for two individuals in our study. PMG is often described as the most frequent malformation of cortical development. However, the true prevalence is unknown or referred to Leventer *et al.*’s study,^1^ where PMG accounts for 16% of all MCD in children. In their study, Leventer *et al.* included all children referred to the major paediatric referral centre for children with neurologic dysfunctions in the state of Victoria, with a population of 4.4 million. Their study included 109 patients with MCD under the age of 19 years. In our study, we included all children below the age of 18 years with PMG living in Stockholm County. We could calculate that the PMG prevalence in Stockholm was 2.3 per 10 000 children.

Even though PMG is clearly defined as a brain malformation with excessive number of abnormally small gyri, in clinical practice PMG is a diagnosis not always easy to convincingly confirm due to MRI imaging quality. In addition, PMG could be visible in different stages of cerebral development. However, the last decade’s development of MRI technics has improved PMG diagnosis and differential diagnoses from radiologically similar pathologies. In addition, attempts to better classify PMG have been ongoing for more than 20 years. Hayashi *et al.*^22^ suggested in 2002 at least four PMG subtypes. A comprehensive classification was done by Barkovich in 2005 with the latest update in 2012. During recent years, the mostly often used classification is the one by Leventer *et al.*^2^, where PMG is divided into 6 topographic patterns and 13 morphological subtypes.

We found it more explicit to define peri-sylvian PMG as being limited to the peri-sylvian cortex. Several cases with extended PMG in the frontal lobe and peri-sylvian area but with anterior-posterior severity gradient were classified as frontal PMG. The most common pattern of PMG reported by several studies is bilateral PMG, predominating peri-sylvian.^9,22,23^ In contrary to other studies, the PNH-PMG group was the most common PMG pattern in our study. This could have several explanations. In the PNH-PMG group, we included all individuals with PNH, even if the amounts of heterotopias were small. In addition, most of our MRI protocols were designed for paediatric patients. Furthermore, image quality has improved over the years, and a majority of our patients had imaging from 3 T scanning. Many patients had repeated imaging, exemplifying the effect of brain maturation on image contrast between grey and white matter, which makes it easier to detect the true extent of PMG, grey matter heterotopias as well as associated findings at different ages. Therefore, if we would have added PNH-PMG cases to the peri-sylvian group, it would have been the largest. In our cohort, bilateral and symmetric was a trend with a relative even distribution. When looking at peri-sylvian PMG, bilateral (*n* = 53) with a right-sided severity was more common than unilateral (*n* = 5), similar to other studies. The lobe most affected was the frontal lobe, followed by the peri-sylvian region that often extended mostly frontal but also into adjacent lobes. PMG was less common in the occipital lobes and along the inter-hemispheric fissure.

The aetiology of PMG is heterogenous and could be caused by several genetic and non-genetic factors. The number of genes associated with PMG is constantly increasing, and today, at least 50 genes are associated with PMG. Pathogenic or likely pathogenic variants were described in 20% of 123 studied individuals with PMG by Stutterd *et al*.^9^ Our study revealed an even higher proportion of genetic aetiologies in PMG, counting for 32% of cases for the whole cohort and 39% among the genetically tested individuals. That could be explained by the rapidly increasing number of genes associated with PMG and the availability of more advanced genetic testing.

Infections, especially congenital CMV, are known to cause PMG. CMV can be asymptomatic during first months of life, and the diagnosis can thus be difficult to confirm beyond the neonatal period, unless there is access to neonatal blood samples. Brain imaging abnormalities in congenital CMV can vary widely—microcephaly, intracranial calcifications, ventriculomegaly, white matter abnormalities and migration disorders.^24,25^ Among 30 individuals with congenital CMV infection, only 7 (23%) had signs suggestive of PMG.^25^ In the study by Manara *et al.*,^26^ 6 of 14 (42%) patients with symptomatic congenital CMV had PMG. In a study by de Vries *et al.*,^27^ among 11 children with congenital CMV 4 (36%) had peri-sylvian PMG. Less frequently described is the CMV prevalence in a PMG cohort. Mavili *et al.*^13^ studied 26 patients with PMG. Six patients (23%) had positive CMV serology; however, the study does not state whether the infection was congenital. In our cohort, only three patients had a confirmed congenital CMV infection. In one additional patient, congenital CMV was suspected due to sensorineural hearing loss and microcephaly, but the diagnosis could not be confirmed due to lack of neonatal blood samples. Our study suggests CMV to be a less frequent cause of PMG than was previously reported. However, some patients might hide among the cases with unknown cause as we could not obtain neonatal blood samples and perinatal data from patients born in other countries or outside the Stockholm region. Another explanation could be a low seroprevalence of CMV in women of childbearing age in Sweden, or that the previously reported high number of CMV in PMG cohorts were mostly case studies and PMG prevalence calculated in a CMV cohort.

According to the international consensus recommendations for MCD, microcephaly is defined as OFC ≤2 SD below the mean for age and sex.^3^ In the Leventer *et al.* study of 328 patients with PMG, microcephaly at birth (defined as OFC ≤2 SD) was reported in 20 of 44 patients (45%). Only 3 of 20 patients in whom OFC was available at birth and at later age developed secondary microcephaly. The authors concluded that microcephaly in association with PMG is usually congenital. In contrary, our results showed that only 14% of patients with PMG had congenital microcephaly, but 38% had microcephaly later in life, and at least half of them developed severe secondary microcephaly. Our data suggest that development of secondary microcephaly is not uncommon in individuals with PMG.

In several studies, macrocephaly in individuals with PMG is reported to count for 6–14%, though definition of macrocephaly is not always equally defined.^2,9,23^ In our study, macrocephaly was observed in 16% at birth and in 10% at later age.

Similar to Leventer *et al.*’s study,^2^ the most common presenting symptom leading to neuroimaging and diagnosis of PMG in our study was seizures. PMG has been reported to be a highly epileptogenic malformation. A neuropathological study of brain tissue from PMG patients points to a changed balance in excitatory and inhibitory synaptic ratios.^28^ Basic underlying mechanisms leading to epileptogenesis in ischaemic polymicrogyric cortex differ from epileptogenesis in genetic PMG, which also in addition involves heterogenous molecular mechanisms. Differences in epileptogenic mechanisms in relation to aetiology likely explain the wide spectrum of seizures and epilepsies in PMG. Epilepsy in patients with PMG is reported in a few studies. The highest frequency of epilepsy in PMG was reported by Kuzniecky *et al.*,^29^ where 27 of 32 (87%) of patients with bilateral peri-sylvian PMG had seizures. This might be explained by the study population and that bilateral peri-sylvian PMG could be more epileptogenic. The lowest epilepsy prevalence in a PMG cohort was found by Teixeira *et al*.^30^ In this study, clinical and EEG features in 40 patients (31 below the age of 18 years) with PMG revealed epilepsy in 33% for the whole cohort and 35% for individuals below the age of 18 years. The epilepsy prevalence in the PMG cohort reported by Leventer *et al.*^2^ was 176 of 225 (78%) patients. However, data on the presence of epilepsy in this study were available in two-thirds of the included patients, and thus, the true epilepsy prevalence could be higher or lower than reported. In our population-based PMG cohort, we present an epilepsy prevalence of 55%. This is lower than reported in several previous studies, but similar to the results of Sah *et al.*^31^ in which 10 of 18 individuals with PMG (55%) had epilepsy. Although our data collection was performed retrospectively, we were able to collect data on epilepsy diagnosis, seizure onset, seizure and epilepsy type in all included patients.

In general, many of our results on epilepsy features in individuals with PMG are in accordance with the findings of Shain *et al*.^23^ Among the similarities, we observed was a history of febrile seizures prior to epilepsy development in PMG. This was previously reported only by Shain *et al.*, in 11.5% compared with 11.9% in our study. In our cohort, 13 (12%) patients had febrile seizures as the first presenting seizure. In six of these patients, the presenting seizure type was single or recurrent febrile seizures, prior to unprovoked seizures and development of epilepsy. Due to the young age of many individuals in our cohort, it might be an even more common finding.

The reported age at seizure onset in individuals with PMG varies in different studies. A multi-centre study on PMG associated epilepsy demonstrated a median seizure onset of 3 years and mean of 6 years of age.^23^ In the study by Leventer *et al.*^2^ of PMG, the median seizure onset was 2 years, and the mean age 4.9 years. Both studies included children as well as adults. Compared with previous studies, our cohort demonstrated a younger age at seizure onset, with median of 1.6 years and mean age of 2.5 years. This could be explained by study inclusion criteria as only children below age of 18 years were included in our cohort. Similarly to Leventer *et al.*’s results, our study demonstrated seizure onset within the first year of life in 44% of patients, and earlier seizure onset was observed in individuals with generalized PMG and bilateral peri-sylvian PMG.

We observed a gender difference in the epilepsy group versus the whole PMG cohort. While male dominance constituted almost two-thirds of the whole PMG cohort, there was almost an equal number of females and males in the epilepsy cohort. However, the difference was not statistically significant. These data might indicate that females with PMG could be more prone to develop epilepsy.

The response to ASM in a PMG population is not sufficiently studied. One PMG-associated epilepsy study reports, similar to our study, the number of median used for ASM.^23^ A recent study on long-term outcome of epilepsy in different MCDs showed that 60% of all patients had pharmacoresistant epilepsy.^32^ Authors in the aforementioned study concluded that a unilateral distribution of the MCD was associated with a 3-fold higher probability of achieving epilepsy remission defined as seizure-freedom for at least 5 years. We compared pharmacoresponse in individuals with uni- and bilateral PMG. However, our results could not reveal any significant difference in pharmacoresponse. In tuberous sclerosis complex, a genetic disorder with focal cortical malformations called tubers, up to 85% have epilepsy, and of these, 62% develop pharmacoresistant epilepsy.^33^ We could demonstrate almost as high number of pharmacoresistant epilepsy in our PMG cohort (56%).

Strengths of this study are the population-based study population and the access to publicly available register data. In addition, due to centralized paediatric neurology care in Region Stockholm and digital medical journals, we were able to obtain comprehensive clinical information on all included patients. Other strengths are a low population mobility and long-term patient follow-up at the two paediatric neurology units as these patients are not referred to general practice care for their neurological disorders.

However, there are some limitations in our study. The included patients were selected from radiologically diagnosed PMG and therefore we might have missed cases. To reduce this bias, the primary database search included MRI scans with clinically diagnosed PMG, pachygyria and schizencephaly. At re-evaluation, pachygyria and schizencephaly without PMG were not included in the study cohort. Not all children with mild cognitive impairments undergo brain MRI and are therefore not diagnosed with PMG. Most often, PMG presents in childhood; however, PMG could be diagnosed in adulthood, and the prevalence could thus be higher. Another limitation of our study is its retrospective nature with clinical data collected from medical records.

We are currently working on a follow-up paper with focus on genetic results and correlation among genetic variants, neuroimaging and phenotype.

## Conclusion

The prevalence of PMG in Stockholm County was 2.3 per 10 000 children. Confirmed genetic aetiology was present in one-third of individuals with PMG, higher than previously reported. The most common PMG distribution was in the frontal lobe. Epilepsy occurred in 54% of individuals, and the majority had pharmacoresistant epilepsy. Neurodevelopmental disorders were common and highlight the need for multi-professional care.

## Supplementary Material

fcad213_Supplementary_Data

## Data Availability

Data supporting results of our study are available from the corresponding author upon reasonable request.
