# Supplementary material for: Polymicrogyria: epidemiology, imaging, and clinical aspects in a population-based cohort
Source: Brain Commun. 2023 Aug 11;5(4):fcad213. doi: 10.1093/braincomms/fcad213 (PMC10443657; doi:10.1093/braincomms/fcad213)
Supplement: fcad213_Supplementary_Data [file fcad213_supplementary_data.pdf]

Supplementary Table 1 Genetic aetiologies in our cohort of 109 individuals with polymicrogyria

Single gene variants		Chromosomal aberrations
Pathogenic/probably pathogenic	VUS	
ACTB	*ADGRG1*	dup13q31.3q34
AKT3	*SLC6A17*	del22q11.21
AP1S2	*AHDC1*	delXp12pter
ASXL3	*ATP1A3*	del1q43qter
BBS2	*GRIA3*	der(6)t(6;13)(p25;q32)del6(p25pter),dup13(q32qter)
COL4A1	*NPHP1*	del1p36
DEPDC5	*NPRL3, n*=2	
DYNC1H1, n=3	*PTPN23*	
GRIN2B	*ROBO1*	
OFD1	*SCN3A*	
PEX1	*SDHA*	
PEX2	*SON*	
PIK3CA	*TSC2*	
PIK3R2, n=2		
POMGNT1, n=3		
RAB3GAP1, n=2		
SCN2A		
SNAP29		
TSC1		
TUBA1A, n=2		
WDR62, n=2		

VUS - variants of uncertain significance.

Supplementary Table 2 The frequency of neurodevelopmental disorders in relation to epilepsy

	**Without epilepsy, *n*=50**		**With epilepsy, *n*=59**		
**Neurodevelopmental disorders**	***n***	**%**	***n***	**%**	**p***
Cognition					
Development delay	8	16	9	15.3	0.92
Intellectual disability	23	46	35	59.3	0.17
Learning difficulties	<5	N/A	<5	N/A	1
ASD	11	22	5	8.5	0.06
ADD/ADHD	9	18	7	11.9	0.37
Motor function					
Cerebral palsy	14	28	22	37.3	0.3
Motor disorder	16	32	10	17	0.07
Hypotonia	<5	N/A	5	8.5	1

*Chi2-test or Fisher exact. ASD – autism spectrum disorder; ADD – attention deficit disorder; ADHD – attention deficit hyperactivity disorder; N/A - not applicable.

Supplementary Table 3 The frequency of epilepsy in relation to polymicrogyria distribution

	**Without epilepsy, *n*=50**		**With epilepsy, *n*=59**		
**PMG distribution**	***n***	**%**	***n***	**%**	**p***
Frontal	33	66	44	74.6	0.33
Parietal	20	40	20	33.9	0.51
Temporal	16	32	21	35.6	0.69
Occipital	7	14	5	8.5	0.38
Global	9	18	8	13.6	0.52
Interhemispheric	10	20	10	17	0.68
Perisylvian	24	48	34	57.6	0.32

*Chi2-test or Fisher exact. PMG - polymicrogyria
